# A Comparative Study Between Coblation-Assisted Tonsillectomy and Conventional Dissection and Snare Tonsillectomy

**DOI:** 10.7759/cureus.68281

**Published:** 2024-08-31

**Authors:** Akshita Goyal, Paresh Chavan, Vinod Shinde, Gundappa Mahajan, Mayur Ingale

**Affiliations:** 1 Department of Otolaryngology, Head and Neck Surgery, Dr. D. Y. Patil Medical College, Hospital, and Research Centre, Dr. D. Y. Patil Vidyapeeth (Deemed to be University), Pune, IND

**Keywords:** dissection and snare, comparison, efficacy, conventional, tonsillectomy, coblation

## Abstract

Background

Tonsillitis is a vastly prevalent disease, accounting for the majority of outpatient visits. The dissection and snare method has been the predominant approach for tonsillectomy for centuries. Coblation-assisted tonsillectomy offers advantages such as faster healing, shorter surgery duration, minimal blood loss, and fewer postoperative complications. Therefore, a study was conducted to evaluate the distinctions and compare the efficacy of traditional dissection and coblation-assisted tonsillectomy.

Materials and methods

Patients were divided into two groups: Group I was operated on using the conventional method, and Group II was operated on using the coblation method. Both groups were then assessed for intraoperative time, intraoperative bleeding, postoperative pain, and postoperative complications.

Results

Coblation-assisted tonsillectomy patients had a significantly shorter mean duration for the procedure and significantly lower blood loss in comparison to the conventional method. There were no statistically significant variations in the incidence of postoperative complications. Coblation-assisted tonsillectomy patients experienced considerably higher pain scores on various postoperative days.

Conclusion

Coblation-assisted tonsillectomy had the added advantage of reduced intraoperative blood loss, shorter surgical time, and faster recovery postoperatively. Coblation-assisted tonsillectomy can be considered an effective alternative to conventional methods. However, it's important to consider factors such as cost-effectiveness and surgeon experience. Further research involving larger sample sizes and longer follow-up periods could yield more insightful knowledge of the outcomes of these two tonsillectomy techniques.

## Introduction

Tonsillitis is a prevalent medical condition characterized by the inflammation of the tonsils, typically resulting from bacterial or viral infections [1]. The tonsils, which are lymphoid tissue clusters close to the respiratory and digestive system entrances, are essential to our immune system's function. They act as the main barrier, triggering the immune system's defense against ingested or breathed microorganisms. Common symptoms of tonsillitis include a sore throat, inflamed and swollen tonsils, pain during swallowing, fever, cough, headache, fatigue, chills, swollen lymph nodes in the neck, and ear or neck pain. Tonsillitis, often linked with adenoid hypertrophy, is a prevalent condition observed in pediatric patients and occasionally in juveniles and adults as well. Surgical intervention, typically through tonsillectomy or adenotonsillectomy, is recommended when symptoms include a disabling sore throat that interferes with normal activities, recurrent infections despite medical treatment, peritonsillar abscess, unilateral enlargement of tonsils raising suspicion of malignancy, or obstructive sleep apnea [2]. The dissection and snare method has been the predominant approach for tonsillectomy worldwide. A newer technique, coblation-assisted tonsillectomy, utilizes radio frequency to remove tissue and ensure hemostasis [3]. A study was conducted to evaluate the distinctions between traditional dissection-snare tonsillectomy and coblation-assisted tonsillectomy in terms of intraoperative time, intraoperative bleeding, postoperative complications, and postoperative pain, considering the various surgical techniques available for this procedure.

## Materials and methods

A prospective randomized study was conducted on 50 patients admitted to the Department of Otorhinolaryngology at Dr. D. Y. Patil Medical College, Hospital, and Research Centre, a tertiary hospital in Pune, India.

In a study carried out by K. Muthubabu et al. [4], the mean duration was 26.67 (+/-6.65) and 32.83 (+/-7.37) intraoperative time in dissection and snare and coblation tonsillectomy, respectively. By entering this data with a 95% confidence interval and a 5% significance level, the calculated sample size was 42. However, in this study, we included 50 participants. The software used was WinPepi version 11.38 (J. H. Abramson, Brixton Health, United Kingdom). The age group of the patients ranged from seven years to 40 years. Patients with symptomatic tonsillar hypertrophy with or without adenoid hypertrophy who were willing to give consent for surgery were included. Children below seven years of age were excluded. Patients with conditions like bleeding disorders or asthma, peritonsillar abscess, chronic systemic illness, hemoglobin less than 10 g/dL, pregnancy and lactation, history of tonsillitis three weeks prior to surgery, immunocompromised status, and unwillingness to undergo surgery were excluded from the study.

Using a table of random numbers, 50 patients with a complete diagnosis of chronic tonsillitis with or without adenoid hypertrophy were split into two groups of 25 participants each. Patients assigned to Group I had a traditional tonsillectomy using the dissection and snare technique whereas patients in Group II had a tonsillectomy with coblator assistance. The surgery was carried out by the same surgeon to avoid bias. The amount of intraoperative bleeding, intraoperative surgical time, postoperative complications, and days taken to return to a normal diet were assessed in all patients postoperatively. Postoperatively, all patients were given one day of injectable analgesia followed by three days of oral analgesics. Postoperative pain was assessed using a simple descriptive pain scale as a subjective indicator for the severity of pain on postoperative days (PODs) 0, one, and three (Table 1).

**Table 1:** Simple descriptive pain scale

Pain score	Severity
10	Unable to move
9	Severe
8	Intense
7	Unmanageable
6	Distressing
5	Distracting
4	Moderate
3	Uncomfortable
2	Mild
1	Minimal
0	No pain

The mean responses were identified and tabulated. The p-value was computed to compare the two methods, and the results were determined based on the statistical analysis. Additionally, a comprehensive review of various studies was conducted to align the findings with existing literature and statistical data.

## Results

Group I experienced a substantially greater mean blood loss in milliliters than Group II, with values of 31.2 ml and 16.2 ml, respectively. Group I had a standard deviation of 6.8 ml, while Group II had a standard deviation of 4 ml. This p-value of this comparison was less than 0.001, which means that there was a statistically significant difference between the two groups (shown by the letter "S") (Table 2).

**Table 2:** Distribution pattern depending on the intraoperative blood loss in patients (in ml) SD: standard deviation; ml: milliliters; S: statistically significant difference is present as the p=value is less than 0.05

Blood loss (ml)	Group I	Group II	p-value
Mean	31.2	16.2	1.29 × 10⁻¹² (S)
SD	6.8	4	

With mean procedure times of 46.8 minutes and 29.2 minutes, respectively, Group I's mean procedure time was much longer than Group II's. Group II's standard deviation was 6.2 minutes, while Group I's was 17 minutes. The p-value of the comparison was 0.0003, signifying a statistically significant difference (shown by an "S") between the two groups (Table 3).

**Table 3:** Distribution depending on intraoperative time taken SD: standard deviation; S: statistically significant difference present as the p-value is less than 0.05

Time (minutes)	Group I	Group II	p-value
Mean	46.8	29.2	0.0003 (S)
SD	17	6.2	

Group I and Group II had differing pain levels at various PODs. Group I had a mean pain score of 3.2 on POD 0 with a standard deviation (SD) of 1.1, whereas Group II had a mean pain score of 5.4 with an SD of 0.9. In Group I, the mean pain score dropped to 2.1 (SD=0.7) on POD three, while in Group II, it reduced to 2.9 (SD=0.7). By POD five, Group I's mean pain score dropped to 1.3 (SD = 0.5) and Group II's to 1.9 (SD = 0.7). POD 0, POD three, and POD five all had statistically significant p-values (p < 0.001, p = 0.0001, and p = 0.001, respectively) (Table 4).

**Table 4:** Distribution depending on the pain score on various postoperative days POD: postoperative day; SD: standard deviation; S: statistically significant difference present for all three postoperative days as the p-value is less than 0.05

Pain score	Group I		Group II		
	Mean	SD	Mean	SD	p-value
POD 0	3.2	1.1	5.4	0.9	<0.001 (S)
POD 3	2.1	0.7	2.9	0.7	0.0001 (S)
POD 5	1.3	0.5	1.9	0.7	0.001 (S)

In terms of complications, Group I had six cases, while Group II had two cases. The majority of cases, 19 in Group I and 23 in Group II did not experience any complications. The p-value for this comparison was 0.06, indicating no statistically significant difference between the two groups (Table 5).

**Table 5:** Incidence of postoperative complications among the study population NS- no statistically significant difference is present as the p-value is greater than 0.05

Complications present in a number of patients	Group I	Group II	p-value
Yes	6	2	0.06 (NS)
No	19	23	
Total	25	25	

## Discussion

Tonsillectomy is a commonly performed procedure globally, and this study seeks to compare a traditional, widely used surgical method with a newer alternative.

Based on the present study, Group II exhibited significantly lower mean blood loss during the procedure compared to Group I. This finding suggests that coblation-assisted tonsillectomy may lead to reduced intraoperative bleeding compared to conventional tonsillectomy. In particular, Group I saw a considerably larger mean blood loss (31.2 ml) as opposed to Group II (16.2 ml). This result highlights a possible benefit of coblation-assisted tonsillectomy in reducing intraoperative bleeding, which may lead to better surgical results, shorter operating times for hemostasis, and potentially quicker patient recovery. Nallasivam et al. concluded in their study that patients who underwent tonsillectomy via the conventional method suffered a larger mean blood loss (43.4 ml) compared to patients who underwent coblation-assisted tonsillectomy (18.7 ml) [5].

Based on the present study, Group II had a significantly shorter mean duration for the procedure compared to Group I. This finding suggests that coblation-assisted tonsillectomy may be associated with shorter intraoperative times, potentially leading to reduced surgical time and anesthesia exposure. This outcome highlights a potential advantage of coblation-assisted tonsillectomies in terms of efficiency, which may benefit both patients and surgical teams by reducing the duration of anesthesia and overall surgical procedure time. Zainon et al., in their report, concluded that the conventional method took a longer operative time (7.2 minutes) in comparison with coblation-assisted cases (4.2 minutes) [6]. In comparing the study done by Rakesh et al., coblation-assisted tonsillectomy took 15 minutes of operative time, whereas the conventional method was only 11 minutes [7]. Noordzij et al., in their study, concluded that there was not a significant difference in intraoperative time for both procedures [8]. In comparison with various studies, while some found the coblation-assisted method to have a shorter mean duration for the procedure, others concluded that it took longer and required surgical expertise.

Based on the present study, the rates of complications associated with both procedures are identical. A study by Karathia et al. showed that only one patient experienced a hemorrhage on the right side on the seventh day (secondary hemorrhage), leading to delayed healing [9]. Postoperative pain scores were found to be much higher in patients who underwent a coblation-assisted tonsillectomy. Mitic et al. observed from their research that over a 10-day period, patients receiving coblation-assisted tonsillectomy experienced less discomfort, returned to normal food and activities more quickly, and used fewer analgesics than those undergoing dissection tonsillectomy [10]. Jat et al., in their study, also concluded that postoperative pain was much higher in patients who underwent tonsillectomy via the conventional method [11].

Patients who underwent coblation-assisted tonsillectomy experienced several advantages compared to those who underwent dissection tonsillectomy. They experienced lower overall morbidity and a detrimental effect on health than those who underwent dissection tonsillectomy [10].

Muthubabu et al., in their prospective study, reached the following conclusions: coblation is a relatively easy technique to perform, providing a bloodless field and minimal tissue damage. The operative time required to perform the coblation method was longer than the dissection method. The longer time did not cause more intraoperative blood loss or increased postoperative pain. The intraoperative blood loss was significantly less in the coblation method than in the dissection and snare methods. The postoperative pain scores were significantly lower on the coblation method, which helps the patient resume their normal activities early [4]. 

Nithya et al. performed a similar study in their tertiary center and observed that coblation-assisted surgery took almost double the time of a cold steel surgery on average. In their study, the average duration of surgery in the coblation group was 55.6 minutes, compared to 34.1 minutes in the cold dissection group. Increased operative time implies more tissue handling, which may result in more postoperative pain [12].

Magdy et al., in comparison with multiple techniques, observed that coblation promises better excision hemostasis than cold tonsillectomy methods while transmitting less heat to the pharynx than electrocautery [13].

In Mostafa et al., a similar study involving 60 patients showed that the coblation technique is associated with less incidence of primary tonsillectomy hemorrhage than the dissection technique but a higher incidence of secondary tonsillectomy hemorrhage. The mean period for returning to a normal diet was significantly shorter in the coblation group; also, the return to normal activity (measured by resuming job activity) was earlier in the coblation group [14].

## Conclusions

In conclusion, the findings indicate that coblation-assisted tonsillectomy offers advantages such as reduced intraoperative blood loss, shorter surgical time, and faster recovery to a normal diet compared to conventional tonsillectomy. Although conventional methods may be preferred for managing postoperative pain, the study underscores the potential benefits of coblation-assisted tonsillectomy in enhancing patient outcomes and expediting recovery. However, it is crucial to take into account individual patient factors and surgical preferences when selecting the appropriate tonsillectomy method. While coblation-assisted tonsillectomy shows advantages in areas like reduced intraoperative blood loss and faster recovery, factors such as cost-effectiveness, surgeon experience, and specific patient considerations should also be carefully considered. Further research involving larger sample sizes and longer follow-up periods could yield more insights into the comparative effectiveness and long-term outcomes of these two tonsillectomy techniques. This will facilitate physicians in making well-informed recommendations that are customized to meet the distinct requirements and preferences of every patient.
